# Case report: multiple systemic disseminated tuberculosis mimicking lymphoma on 18F-FDG PET/CT

**DOI:** 10.1097/MD.0000000000007248

**Published:** 2017-07-21

**Authors:** Shasha Hou, Jie Shen, Jian Tan

**Affiliations:** a Department of Nuclear Medicine, Tianjin Medical University General Hospital, Heping District; b Department of Nuclear Medicine, Tianjin First Central Hospital, TianJin China.

**Keywords:** ^18^F-fluorodeoxyglucose positron emission tomography/computed tomography, lymphoma, maximum standardized uptake value, tuberculosis

## Abstract

**Rationale:**

^18^F-fluorodeoxyglucose positron emission tomography/computed tomography (F-18 FDG PET/CT) has an important role in the diagnosis of various malignancies. However, F-18 FDG can also exhibit intense accumulation in tissues in inflammatory conditions such as active tuberculosis (TB) and sarcoidosis.

**Patient concerns:**

We report a case of a 52-year-old female with irritable cough. CT showed a lung mass with multiple bilateral lung nodules, and sarcoidosis was suspected. F-18 FDG PET/CT was undertaken for the diagnosis and showed intense uptake of FDG in the mass in the lower lobe of the right lung, multiple lymph nodes, liver, and spleen. The maximum standardized uptake value of F-18 FDG was 43.58. This pattern of involvement most likely represents lymphomatous involvement.

**Diagnoses:**

Histopathology suggested tubercular involvement.

**Intervention and outcomes:**

The patient received anti-TB treatment and recovered.

**Lessons:**

Abovementioned extent and distribution of F-18 FDG in tubercular lesion is relatively rare, thus, one must be observant and aware with regards to TB being a strong mimic of lymphoma in endemic regions.

## 1 Introduction

Tuberculosis (TB) is a systemic infectious disease caused by *Mycobacterium tuberculosis* that affects multiple tissues and organs. TB is predominantly characterized by generalized lymph nodes (LNs) involvement. It is often impossible to differentiate between TB and malignant involvement on the basis of imaging studies. Diagnosis of tubercular LN on clinical examination is cumbersome as it often manifests with nonspecific symptoms.

^18^F-fluorodeoxyglucose positron emission tomography/computed tomography (F-18 FDG PET/CT) has become an established tool for cancer evaluation. However, FDG delivers a diagnostic dilemma when distinction between inflammatory^[1,2]^ and malignant lesions is in question as both intensely accumulate F-18 FDG. We present a case of TB disseminated in multiple LNs mimicking lymphoma on F-18 FDG PET/CT. The report describes TB as a valid differential when diagnosis of lymphoma based on pattern of involvement is in question.

## 2 Case presentation

**Figure 1:**
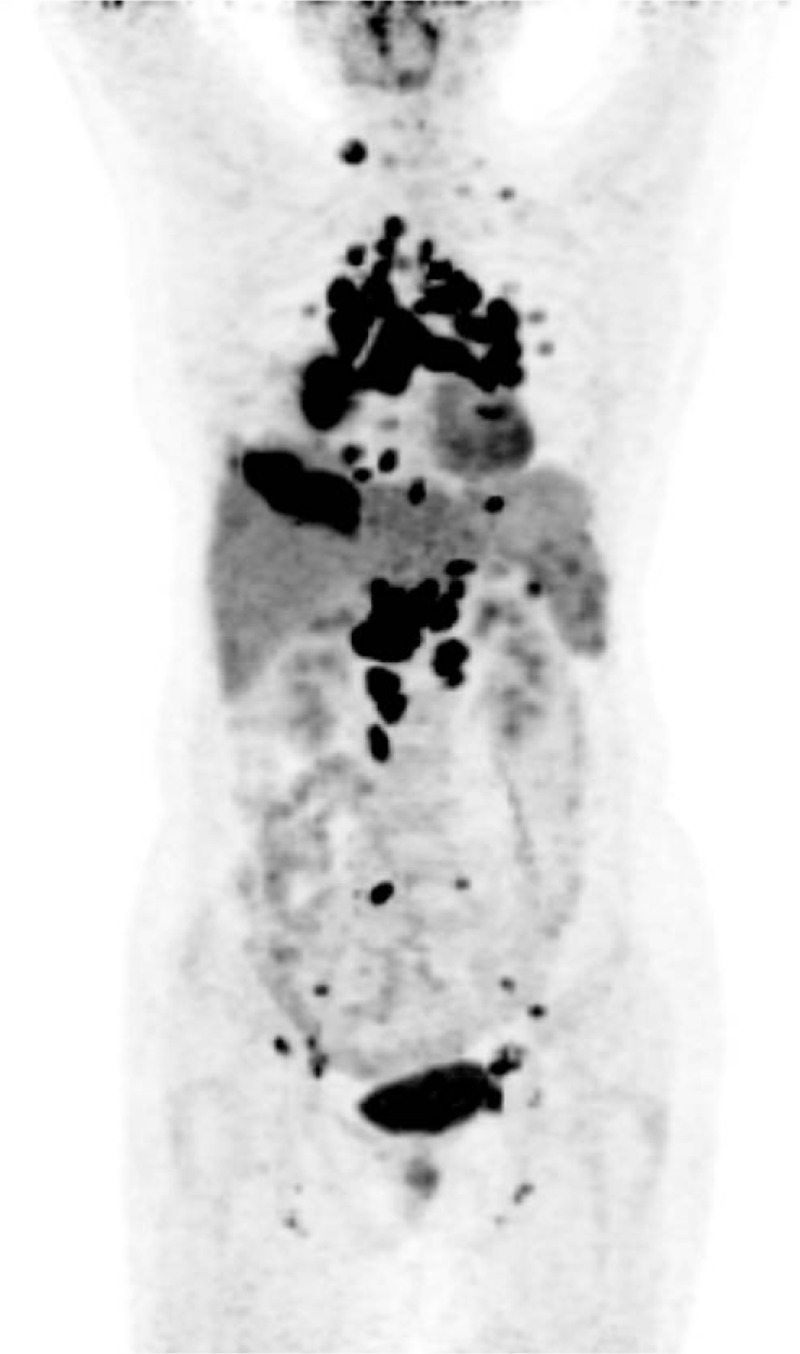
Maximum intensity projection image for F-18 FDG PET/CT showing intense accumulation of F-18 FDG in multiple lymph nodes, lung, liver, and spleen. F-18 FDG PET/CT = ^18^F-fluorodeoxyglucose positron emission tomography/computed tomography, SUV_max_ = maximum standardized uptake.

A 52-year-old female presented to our hospital with irritable cough of duration >1 month, but had no hemoptysis, respiratory distress, fever with chills, diarrhea, fatigue, weight loss, or other nonspecific constitutional symptoms. CT of the chest suggested a right-lung mass with multiple bilateral pulmonary nodules as well as enlarged bilateral hilar and mediastinal LNs. Ultrasound of the abdomen revealed enlarged LNs near the pancreatic head. The level of neuron specific enolase (NSE) in serum was 36.67 (normal range, 0–16.3) ng/mL. The level of erythrocyte sedimentation rate (ESR) and c-reactive protein (CRP) in serum respectively was 52 (0–20 mm/hour) and 5.59 (0–8 mg/L).^99^TC^m^-MDP whole-body bone scintigraphy was normal. Bronchoscopy revealed mucosal chronic inflammation on pathology. The effect of antiinfection treatment was not obvious. For further diagnosis of the lesions, F-18 FDG/PET was undertaken. One hour after intravenous injection of 370 MBq of F-18 FDG, whole-body F-18 FDG PET/CT was done using a Biograph mCT 64 system (Siemens, Hamburg, Germany). The PET/CT scan showed a mass in lower lobe of right lung with intense accumulation of F-18 FDG in multiple LNs, liver, and spleen on maximum intensity projection images (Fig. 1).

**Figure 2:**
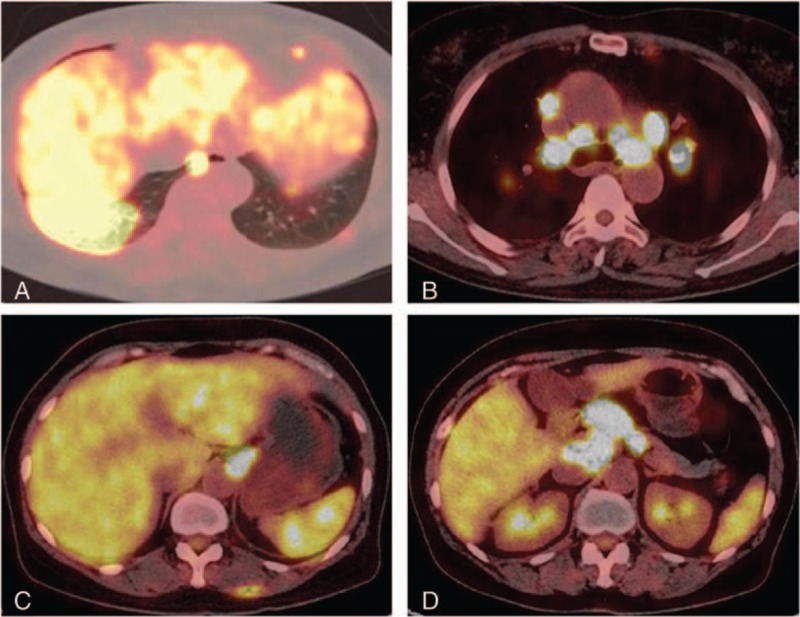
Selected transaxial F-18FDG PET/CT slices of high uptake of F-18 FDG in the mass in the lower lobe of the right lung (SUV_max_ = 20.44), bilateral pulmonary hilar and mediastinal lymph nodes (30.4), and around the lymph nodes near the pancreatic head (43.58). F-18 FDG PET/CT = ^18^F-fluorodeoxyglucose positron emission tomography/computed tomography, SUV_max_ = maximum standardized uptake.

**Figure 3:**
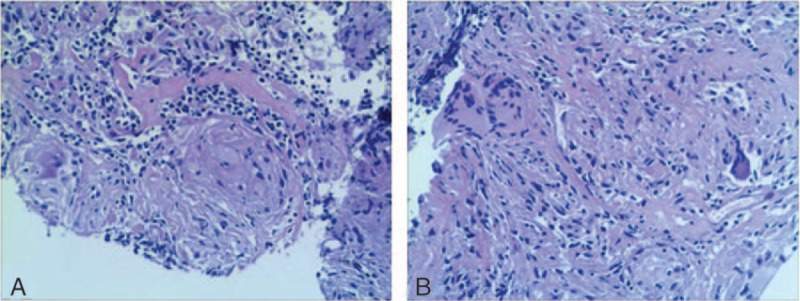
Histopathology of the mass in the lower lobe of the right lung showing caseous necrosis and multinucleated giant cells. The final diagnosis was pulmonary tuberculosis.

An F-18 FDG whole-body scan showed intense accumulation of F-18 FDG in the: mass in the lower lobe of the right lung (Fig. 2A); supraclavicular LNs; bilateral pulmonary hilar and mediastinal LNs (Fig. 2B); bilateral internal mammary LNs (Fig. 2B); paracardial LNs; bilateral costophrenic-angle LNs; abdominal LNs (Fig. 2C, D); retroperitoneum beside the LNs in the abdominal aorta (Fig. 2D); pelvic LNs; bilateral LNs in the space between the gluteal muscles; and bilateral inguinal LNs. In addition, multifocal moderate uptake of F-18 FDG was observed in the liver and spleen (Fig. 2C). PET/CT showed distribution of bilateral pulmonary hilar LNs to be symmetrical. The maximum standardized uptake (SUV_max_) value was 20.44 and 43.58 in the mass in the lower lobe of the right lung and LNs near the pancreatic head, respectively. All lesions showed different degrees of F-18 FDG uptake. These PET/CT findings suggested lymphoma or sarcoidosis, which resulted in diagnostic difficulties. TB is a typical F-18 FDG avid infection. Clinical manifestations of the patient were unremarkable, and the patient had not been diagnosed with an immune system-based disease or TB previously. Laboratory examinations for TB detection (Mantoux test, acid-fast staining, TB antibodies, T-SPOT) were normal. Moreover, the typical signs of TB in hypermetabolic lesions (eg, a well-defined margin and calcification) were absent. Based on the high uptake of F-18 FDG in lesions (SUV_max_ = 43.58), medical history, and imaging findings of our patient, lymphoma was first suspected. To confirm the diagnosis, we undertook aspiration biopsy under CT guidance of the mass in the lower lobe of the right lung. The histopathologic diagnosis was pulmonary TB (Fig. 3). The patient received anti-TB treatment and recovered.

Written informed consent was obtained from the patient for this case report. The consent procedure was approved by the Ethics Committee of the Tianjin Medical University General Hospital.

## 3 Discussion

TB is not uncommon in China, and the number of TB patients is the 2nd highest worldwide. Around 250,000 patients die of TB each year in China.^[3]^ Hence, the medical burden due to TB in China is considerable.

PET can be used to image metabolic differences between normal cells and malignant cells using F-18 FDG.^[4]^ However, false-positive cases are constantly reported because F-18 FDG is absorbed not only by tumor cells but also by inflammatory/infective lesions, such as those affected by TB or sarcoidosis.^[5]^

Generalized lymphadenopathy with increased accumulation of F-18 FDG in pulmonary TB is not rare, which can make the differential diagnosis even more complicated. Studies have shown that LNs are the 2nd most common site of TB infection after the lungs.^[6]^ Differentiation between benign and malignant LNs remains a challenge in diagnostic imaging, especially when active TB is in question, because their treatments are very different. Studies on preoperative staging of lung cancer with F-18 FDG PET/CT, false-positive mediastinal, and hilar LNs are frequently encountered due to tuberculous lymphadenitis (which is prevalent in TB-endemic countries).^[7]^

In our case, pulmonary carcinoma could be excluded on CT. PET/CT showed multiple LNs with increased uptake of F-18 FDG. The diagnosis of TB and sarcoidosis were not considered, especially in the absence of laboratory examinations, medical history of TB, and immune system-based disease. Furthermore, lymphoma also does not exhibit typical symptoms and is usually painless. The diagnosis was confirmed by histopathology. Similar cases were reported where TB mimics lymphoma on F-18 FDG PET/CT.^[8,9]^ The present case manifested with only irritable cough which was neglected by the primary Physician and can be associated with other conditions on F-18 FDG PET/CT.

## 4 Conclusion

TB disseminated to multiple LNs can mimic lymphoma. Upon PET/CT, TB, and lymphoma can show high uptake of F-18 FDG. Therefore, measuring SUV_max_ to differentiate between them is not reliable. Eventhough SUV_max_ cannot reliably distinguish inflammation from malignant process, it is still an effective tool for chalking out therapeutic response.^[10]^ This case study demonstrates the necessity of considering tubercular LNs as a possible differential diagnosis if the patient exhibits subtle symptoms and laboratory tests for TB are negative. An early biopsy for the histopathologic diagnosis is also advisable.

## Acknowledgments

The authors thank the staff of the Department of Nuclear medicine at Tianjin First Central Hospital for providing valuable clinical support.
